# Supplementary material for: Bi-Directional Axial Transmission measurements applied in a clinical environment
Source: PLoS One. 2022 Dec 30;17(12):e0277831. doi: 10.1371/journal.pone.0277831 (PMC9803229; doi:10.1371/journal.pone.0277831)
Supplement: S1 Table — (PDF) [file pone.0277831.s001.pdf]

Appendix 1

Table S1. Discrimination obtained with logistic regression: odds ratios (OR) and areas under the ROC curve (AUC) for BDAT technique on the first population (106 patients).

	all non-traumatic fractures F (N = 27)			Non vertebral fractures NVF (N = 14)		
	AUC [95% CI]	OR [95% CI]	p	AUC [95% CI]	OR [95% CI]	p
unadjusted						
Ct.Th	0.62 [0.48 - 0.74]	1.52 [0.98 - 2.36]	0.058	0.67 [0.45 - 0.79]	1.80 [1.01 - 3.22]*	**0.042**
Ct.Po	0.57 [0.43 - 0.68]	1.30 [0.82 - 2.04]	0.252	0.68 [0.48 - 0.79]	1.86 [1.02 - 3.39]*	**0.040**
VFAS	0.64 [0.52 - 0.72]	1.63 [1.01 - 2.64]*	**0.043**	0.68 [0.54 - 0.81]	1.82 [0.97 - 3.39]	0.055
VA0	0.59 [0.47 - 0.71]	1.41 [0.78 - 2.24]	0.136	0.71 [0.51 - 0.83]	2.25 [1.11 - 4.53]*	**0.021**
unadjusted combinations						
Ct.Po/Ct.Th	0.65 [0.52 - 0.76]	1.74 [1.10 - 2.76]*	**0.017**	0.79 [0.67 - 0.88]	3.11 [1.52 - 6.37]**	**0.001**
Ct.Th	0.66 [0.51 - 0.77]	1.74 [1.07 - 2.83]*	**0.024**	0.79 [0.68 - 0.87]	3.18 [1.40 - 7.21]**	**0.005**
Ct.Po		1.56 [0.93 - 2.61]	0.082		3.39 [1.42 - 8.11]**	**0.005**
adjusted						
Ct.Th	0.62 [0.52 - 0.75]	1.36 [0.84 - 2.20]	0.204	0.75 [0.61 - 0.85]	1.38 [0.74 - 2.59]	0.306
Ct.Po	0.62 [0.50 - 0.73]	1.16 [0.70 - 1.91]	0.566	0.76 [0.64 - 0.83]	1.62 [0.82 - 3.19]	0.155
VFAS	0.63 [0.52 - 0.75]	1.51 [0.86 - 2.64]	0.141	0.72 [0.58 - 0.82]	1.34 [0.63 - 2.85]	0.443
VA0	0.61 [0.47 - 0.74]	1.18 [0.65 - 2.16]	0.574	0.74 [0.56 - 0.84]	1.79 [0.74 - 4.32]	0.187
adjusted combinations						
Ct.Po/Ct.Th	0.66 [0.53 - 0.77]	1.67 [0.95 - 2.96]	0.071	0.81 [0.65 - 0.90]	3.14 [1.26 - 7.82]*	**0.012**
Ct.Th	0.66 [0.54 - 0.77]	1.72 [0.95 - 3.12]	0.067	0.81 [0.72 - 0.89]	3.36 [1.21 - 9.32]*	**0.017**
Ct.Po		1.58 [0.85 - 2.93]	0.141		3.92 [1.32 - 11.65]*	**0.012**

Reference category is non fractured (NF) N = 79. CI confidence interval. ROC receiver operating characteristic, AUC and OR are adjusted for age, BMI and gender; **$p < 0.05$; **$p < 0.01$; ***$p < 0.001$..
