# Supplementary material for: Bi-Directional Axial Transmission measurements applied in a clinical environment
Source: PLoS One. 2022 Dec 30;17(12):e0277831. doi: 10.1371/journal.pone.0277831 (PMC9803229; doi:10.1371/journal.pone.0277831)
Supplement: S2 Table — (PDF) [file pone.0277831.s002.pdf]

Appendix 2

Table S2. Discrimination obtained with logistic regression: odds ratios (OR) and areas under the ROC curve (AUC) for both techniques BDAT and DXA and the second population (87 patients).

	all non-traumatic fractures F (N = 23)			Non vertebral fractures NVF (N = 13)		
	AUC [95% CI]	OR [95% CI]	p	AUC [95% CI]	OR [95% CI]	p
unadjusted parameters						
Ct.Po/Ct.Th	0.65 [0.51 - 0.77]	1.76 [1.05 - 2.93]*	**0.027**	0.78 [0.60 - 0.88]	3.05 [1.44 - 6.46]**	**0.003**
Ct.Th	0.66 [0.52 - 0.78]	1.69 [1.00 - 2.88]*	**0.047**	0.79 [0.67 - 0.91]	2.76 [1.20 - 6.34]*	**0.015**
Ct.Po		1.60 [0.93 - 2.77]	0.084		3.21 [1.33 - 7.72]**	**0.008**
aBMD total	0.70 [0.55 - 0.79]	2.14 [1.19 - 3.84]**	**0.010**	0.74 [0.60 - 0.86]	2.53 [1.19 - 5.40]*	**0.014**
aBMD fn	0.70 [0.52 - 0.81]	2.39 [1.28 - 4.49]**	**0.005**	0.77 [0.62 - 0.89]	3.48 [1.43 - 8.50]**	**0.005**
aBMD spine	0.61 [0.46 - 0.76]	1.49 [0.87 - 2.56]	0.142	0.63 [0.49 - 0.77]	1.69 [0.85 - 3.37]	0.128
adjusted parameters						
Ct.Po/Ct.Th	0.68 [0.54 - 0.79]	1.52 [0.81 - 2.85]	0.186	0.83 [0.71 - 0.91]	2.62 [1.02 - 6.75]*	**0.042**
Ct.Th	0.69 [0.55 - 0.81]	1.50 [0.78 - 2.88]	0.218	0.83 [0.73 - 0.92]	2.33 [0.81 - 6.72]	0.112
Ct.Po		1.49 [0.77 - 2.85]	0.224		3.04 [1.04 - 8.92]*	**0.038**
aBMD total	0.74 [0.58 - 0.83]	2.88 [1.31 - 6.32]**	**0.007**	0.81 [0.64 - 0.92]	3.33 [1.10 - 10.04]*	**0.029**
aBMD fn	0.74 [0.62 - 0.85]	2.64 [1.21 - 5.77]*	**0.013**	0.82 [0.71 - 0.91]	3.48 [1.11 - 10.91]*	**0.029**
aBMD spine	0.69 [0.54 - 0.80]	1.72 [0.87 - 3.42]	0.111	0.78 [0.65 - 0.87]	1.62 [0.64 - 4.07]	0.296

Reference category is non fractured (NF) N = 64. CI confidence interval. ROC receiver operating characteristic, AUC and OR are adjusted for age, BMI and gender; **$p < 0.05$; **$p < 0.01$; ***$p < 0.001$.
