# Supplementary material for: Bi-Directional Axial Transmission measurements applied in a clinical environment
Source: PLoS One. 2022 Dec 30;17(12):e0277831. doi: 10.1371/journal.pone.0277831 (PMC9803229; doi:10.1371/journal.pone.0277831)
Supplement: Attachment — Submitted filename: review-D-22-03874.pdf [file pone.0277831.s003.pdf]

REVIEW

The submitted manuscript aims at evaluating the performance of a bidirectional axial transmission (BDAT) device to assess fracture risk in a clinical environment. The proposed approach relies on the measurements of ultrasound guided waves, which have been proven feasible to extract cortical bone quality markers, such as cortical thickness and porosity. In this cross-sectional study, the authors built on earlier results (by the same group, in part, *e.g.*, Refs. [32, 35–37]) but extended their analysis by accounting for two further ultrasound parameters (*i.e.*, the velocity of the first arriving signal and the velocity of the zero-order antisymmetric guided mode). In addition, the measurements were now performed by hospital operators rather than experimented physicists to get closer to a routine clinical environment. Although such kind of studies are promising to improve the identification of patients at high risk of fracture (with respect to DXA) using a portable, low-cost, and radiation-free device, I have several concerns with respect to the manuscript, which prevent its publication in the proposed form. These are highlighted below:

Q_1 A first concern is related to the **abstract**. PlosOne being a journal of multidisciplinary nature oriented to a rather broad audience, I do not think that the terms "velocities" are clear enough at this stage, especially because the authors only mentioned the term BDAT so far, and that the expression "ultrasound parameters" arrives much later. Please clarify this.

Q_2 A second important concern is related to the overall inconsistency of the reported patient numbers belonging to the different categories, as well as other misleading values:

- In the abstract, the authors mention 107 patients, but then the study is conducted on 106 patients only (**line 74**).
- Likewise, the abstract states that the BMI ranges from 17 to 47 kg.m^{-2}, but then the authors write 16 to 47 kg.m^{-2} (**line 69**).
- In the same way, the authors mention 14 patients with non-vertebral fractures (**lines 169–172**): 9 hip fractures, femoral neck ($n = 8$), pelvis ($n = 2$), pertrochanter ($n = 1$) as well as 4 others sites, humerus ($n = 2$), elbow ($n = 1$) and shoulder ($n = 1$) . . . neither $9 + 4 = 14$ nor $8 + 2 + 1 + 2 + 1 + 1 = 14$?
- The study is conducted on 27 fractured patients (for the population comprising 106 patients), but the discussion states 28 fractured patients (**line 283**).
- In **line 74**, it is not clear either whether the "minus two exclusion cases" are the two patients with traumatic fracture.
- Given the important number of reported tables, the statement regarding "p-values decrease from about 0.2 to 0.1 between the second population and the first one" (**lines 291–282**) is far from obvious. The authors should recall the corresponding table to guide the reader.

Q_3 A third concern is related to the applied methodology, the organization of the reported results, and the discussion:

- The first paragraph of the Measurements section (**lines 88–99**) is nearly copy-pasted from an earlier study by the same group (Ref. [37]) and should be rephrased.

- The statements on the *Proj* and *Norm* functions (**lines 100–107**) are very partial and require an accurate knowledge of their previous works (*e.g.*, Refs. [32, 35–37]). This would not be so dramatic at this stage, except that the authors mention this again in the discussion as a perspective of improvement (**lines 286–288**). Overall, they should either tell us more about this (by including some formula and/or dispersion curves for instance) or remove the statement in the discussion.
- In the abtract and the results, the authors decided to put forward the fracture discrimination analysis performed using the PLS-LOOCV, and put the results related to the so-called "classical statistical analysis" (binomial logistic regression analysis) in the appendix. This deliberated choice would not be so problematic in practice, but surprisingly the authors mostly comment on the latter in the discussion (*i.e.*, main findings), while the added value of the former is never discussed (**lines 238–243**).
- Likewise, at the end of the introduction, the authors stated that one of the contribution of the present study is the measurement of two velocities (**lines 62–64**), in addition to the two cortical bone parameters (thickness and porosity). Nevertheless, the added value of measuring as well these two velocities is barely addressed in the manuscript, and neither discussed in the light of former results (*e.g.*, Ref. [32]).
- Based on the significant Spearman's correlations reported in Table 2, the authors conducted their discrimination analysis by using a systematic adjustment for cofounder variables age, BMI and gender (**lines 199–201**). Although the adjustment for age and BMI is straightforward (as done in Ref. [37]), what is the rationale behind the adjustment for gender? Please comment on this.
- In the same way, once the adjustment has been proposed, what is the rationale in keeping the results for unadjusted variables in Tables 5 and 6? The manuscript could gain in clarity by presenting the results in a more concise manner.
- Partly based on Figure 2, one understands that fracture is likely to happen if the cortical thickness decreases and the porosity increases. But, mathematically, what is the rationale in studying the ratio Ct.Po/Ct.Th in the statistical analysis, and to what extent is that different from combining both parameters or studying Ct.Th/Ct.Po for instance ? Please comment on this.
- Following this statement, in Figure 2, apart from providing μ-CT cross-sections from *ex vivo* human bone samples for comparison, it would be also valuable for the reader to show the agreement between measured and modeled dispersion curves for some specific Ct.Th-Ct.Po values.
- QUS approaches are often seen as complementary to DXA, so why not incorporating as well combinations of parameters retrieved both from BDAT and DXA in the analysis (as done in Ref. [37])?
- In the last paragraph of the discussion, the authors should properly separate the limitations of the study from the perspectives. As a further limitation, the authors should also indicate that DXA measurements were performed on skeletal sites that are different from the BDAT ones (*i.e.*, one-third distal radius). Overall, the discussion is quite poor and has room for improvement.

Q_4 A last concern is related to the overall **presentation** of the manuscript. It contains many typos, misleading statements, and organization burden, thereby being far from reaching

the acceptable standard for a scientific article. It is a pity because the reported results would be worth being spread to the community, if presented adequately. The authors must drastically review the manuscript. A far from exhaustive list is provided below:

- In the **abstract**, "One hundred and seven ...". The term "patients" is missing.
- **Line 8**: "Even if" should be corrected to "Although".
- **Line 23**: "image processing techniques propose to extract" should be corrected to "image processing techniques allow extracting".
- **Line 29**: "[11].Note" should be corrected to "[11]. Note". Please check elsewhere in the manuscript.
- **Line 32**: "are" should be corrected to "is".
- **Lines 43–45**: The statement on machine learning is disconnected from the former and subsequent sentences.
- **Line 80**: "approved 24.01.2017" should be corrected to "approved on the 24.01.2017".
- **Line 80**: "is achieved" should be corrected to "are achieved".
- **Line 125**: "BMD" should be corrected to "aBMD".
- **Line 126**: "values corresponds" should be corrected to "values correspond".
- **Line 176**: "difference were" should be corrected to "differences were".
- **Lines 186–188**: The statements on the p-value and the VF group should be done after introducing Table 3. Moreover, the latter is introduced before Table 2.
- **Line 209**: "one can observed that the confidence interval are larger" should be corrected to "one can observe that the confidence intervals are larger".
- **Line 213**: "population 1" is misleading as it was never called like this before.
- **Line 213**: "will discussed" should be corrected to "will be discussed".
- **Line 213**: "non-fractured and fractures" should be corrected to "non-fractured and fractured".
- In **Table 3**, the statement on the p-values in the legend does not make sense. By the way, this table has room for improvement, as many elements are repeated twice.
- **Line 228**: The abbreviation "aBMD fn" has never been introduced.
- **Line 258**: "lower the DXA" should be corrected to "lower than the DXA".
- **Line 268**: The statement about the multiscale homogenization (Ref. [45]) is disconnected or should be explained better.
- **Line 271**: "has" should be corrected to "have".
- **Line 273**: "can be evaluate" should be corrected to "can be evaluated".
- **Lines 292–293**: The statement about cortical bone attenuation should be developed further or removed. Indeed, attenuation seems to be somehow already accounted for in the signal processing to "enhance" the dispersion curves extraction. Incorporating attenuation in the waveguide model would be of course interesting too, but this is not trivial (at least analytically), so please provide some more information thereon and how it may provide an added value towards fracture risk assessment.

- In **Tables 5 and 6**, I do think that the term “linear” should be corrected to “logistic”.
- Please unify the **references**, as some of them are provided with complete journal names and some others with abbreviated ones, etc.
