# Supplementary material for: Bi-Directional Axial Transmission measurements applied in a clinical environment
Source: PLoS One. 2022 Dec 30;17(12):e0277831. doi: 10.1371/journal.pone.0277831 (PMC9803229; doi:10.1371/journal.pone.0277831)
Supplement: Attachment — Submitted filename: Herne_response.pdf [file pone.0277831.s004.pdf]

REVIEWERS' COMMENTS:

Reviewer 1

The submitted manuscript aims at evaluating the performance of a bidirectional axial transmis- sion (BDAT) device to assess fracture risk in a clinical environment. The proposed approach re- lies on the measurements of ultrasound guided waves, which have been proven feasible to extract cortical bone quality markers, such as cortical thickness and porosity. In this cross-sectional study, the authors built on earlier results (by the same group, in part, e.g., Refs. [32, 35–37]) but extended their analysis by accounting for two further ultrasound parameters (i.e., the velocity of the first arriving signal and the velocity of the zero-order antisymmetric guided mode). In addition, the measurements were now performed by hospital operators rather than experimented physicists to get closer to a routine clinical environment. Although such kind of studies are promising to improve the identification of patients at high risk of fracture (with respect to DXA) using a portable, low-cost, and radiation-free device, I have several concerns with respect to the manuscript, which prevent its publication in the proposed form. These are highlighted below:

Q1 A first concern is related to the abstract. PlosOne being a journal of multidisciplinary nature oriented to a rather broad audience, I do not think that the terms "velocities" are clear enough at this stage, especially because the authors only mentioned the term BDAT so far, and that the expression "ultrasound parameters" arrives much later. Please clarify this.

The following sentence has been added at the beginning of the Abstract:

> Patients at risk of fracture are currently detected using the current X-Ray gold standard DXA (Dual XRay Absorptiometry). Different alternatives, such as 3D X-Rays, Magnetic Resonance Imaging or Quantitative Ultrasound (QUS) devices, have been proposed, the latter having advantages of being portable and sensitive to mechanical and geometrical properties.

Q2 A second important concern is related to the overall inconsistency of the reported patient numbers belonging to the different categories, as well as other misleading values:

• In the abstract, the authors mention 107 patients, but then the study is conducted on 106 patients only (line 74).

The corrected sentence in the abstract is

> One hundred and **six patients** (**81** women, 25 men) from Marien Hospital and St. Anna Hospital (Herne, Germany) were included in this study.

• Likewise, the abstract states that the BMI ranges from 17 to 47 kg.m^{-}2, but then the authors write 16 to 47 kg.m^{-}2 (line 69).

The correct value, 16 kg.m^{-2}, has been modified in the abstract.

• In the same way, the authors mention 14 patients with non-vertebral fractures (lines 169–172): 9 hip fractures, femoral neck (n = 8), pelvis (n = 2), pertrochanter (n = 1) as well as 4 others sites, humerus (n = 2), elbow (n = 1) and shoulder (n = 1) . . . neither 9 + 4 = 14 nor 8 + 2 + 1 + 2 + 1 + 1 = 14 ?

The corrected sentence is

> Among the 106 patients for whom the measurements were successful, 79 belongs to the control group without fracture (NF), 27 with non-traumatic fractures (F) corresponding to 13 patients with vertebral fractures (VF) and 14 with non vertebral fractures (NVF) associated

with the following sites: **10** hip fractures, femoral neck (n = **7**), pelvis (n = 2), pertrochanter (n = 1) as well as 4 others sites, humerus (n = 2), elbow (n = 1) and shoulder (n = 1).

• The study is conducted on 27 fractured patients (for the population comprising 106 patients), but the discussion states 28 fractured patients (line 283).

The correct value **27** has been modified in the discussion.

• In line 74, it is not clear either whether the "minus two exclusion cases" are the two patients with traumatic fracture.

The expression "minus two exclusion cases" has been replaced by "minus two traumatic fractures".

• Given the important number of reported tables, the statement regarding "p-values decrease from about 0.2 to 0.1 between the second population and the first one" (lines 291–282) is far from obvious. The authors should recall the corresponding table to guide the reader.

The sentence has been removed.

Q3 A third concern is related to the applied methodology, the organization of the reported results, and the discussion:

The different parts have been modified following reviewer's comments.

• The first paragraph of the Measurements section (lines 88–99) is nearly copy- pasted from an earlier study by the same group (Ref. [37]) and should be rephrased.

The paragraph have been modified.

• The statements on the Proj and Norm functions (lines 100–107) are very partial and require an accurate knowledge of their previous works (e.g., Refs. [32, 35–37]). This would not be so dramatic at this stage, except that the authors mention this again in the discussion as a perspective of improvement (lines 286–288). Overall, they should either tell us more about this (by including some formula and/or dispersion curves for instance) or remove the statement in the discussion.

Figure 1 with three typical examples, one per group NF, VF and NVF, of dispersion curves, derived from *Norm* functions and *Proj* functions has been added.

• In the abtract and the results, the authors decided to put forward the fracture discrimination analysis performed using the PLS-LOOCV, and put the results related to the so-called "classical statistical analysis" (binomial logistic regression analysis) in the appendix. This deliberated choice would not be so problematic in practice, but surprisingly the authors mostly comment on the latter in the discussion (i.e., main findings), while the added value of the former is never discussed (lines 238–243).

The discussion has been modified.

• Likewise, at the end of the introduction, the authors stated that one of the contribution of the present study is the measurement of two velocities (lines 62–64), in addition to the two cortical bone parameters (thickness and porosity). Nevertheless, the added value of measuring as well these two velocities is barely addressed in the manuscript, and neither discussed in the light of former results (e.g., Ref. [32]).

The discussion has been modified.

• Based on the significant Spearman's correlations reported in Table 2, the authors conducted their discrimination analysis by using a systematic adjustment for co- founder variables age, BMI and gender (lines 199–201). Although the adjustment for age and BMI is straightforward (as done in Ref. [37]), what is the rationale behind the adjustment for gender? Please comment on this.

The following reference has been added:

Ravindrarajah R, Hazra NC, Charlton J, Jackson SHD, Dregan A, Gulliford MC. Incidence and mortality of fractures by frailty level over 80 years of age: cohort study using UK electronic

health records. BMJ Open. 2018 Jan 21;8(1):e018836. doi: 10.1136/bmjopen-2017-018836.

• In the same way, once the adjustment has been proposed, what is the rationale in keeping the results for unadjusted variables in Tables 5 and 6? The manuscript could gain in clarity by presenting the results in a more concise manner.

Unadjusted and adjusted values have been proposed in Appendix (tables 5 and 6) as reference following the classical way of presenting statistical results. Result paragraph has been modified.

• Partly based on Figure 2, one understands that fracture is likely to happen if the cortical thickness decreases and the porosity increases. But, mathematically, what is the rationale in studying the ratio Ct.Po/Ct.Th in the statistical analysis, and to what extent is that different from combining both parameters or studying Ct.Th/Ct.Po for instance ? Please comment on this.

The two ratios Ct.Po/Ct.Th and Ct.Th/Ct.Po have been tested. The first one provided the best results..

• Following this statement, in Figure 2, apart from providing μ-CT cross-sections from *ex vivo* human bone samples for comparison, it would be also valuable for the reader to show the agreement between measured and modeled dispersion curves for some specific Ct.Th-Ct.Po values.

Agreement between modeled dispersion curves for three specific Ct.Th-Ct.Po values are now shown in Fig. 1.

• QUS approaches are often seen as complementary to DXA, so why not incorporating as well combinations of parameters retrieved both from BDAT and DXA in the analysis (as done in Ref. [37])?

Combination of DXA and BDAT parameters has been added in a new column in Table 4.

• In the last paragraph of the discussion, the authors should properly separate the limitations of the study from the perspectives. As a further limitation, the authors should also indicate that DXA measurements were performed on skeletal sites that are different from the BDAT ones (i.e., one-third distal radius). Overall, the discussion is quite poor and has room for improvement.

The discussion has been modified.

Q4 A last concern is related to the overall presentation of the manuscript. It contains many typos, misleading statements, and organization burden, thereby being far from reaching the acceptable standard for a scientific article. It is a pity because the reported results would be worth being spread to the community, if presented adequately. The authors must drastically review the manuscript. A far from exhaustive list is provided below:

• In the abstract, "One hundred and seven . . .". The term "patients" is missing.

done

• Line 8: "Even if" should be corrected to "Although".

done

• Line 23: "image processing techniques propose to extract" should be corrected to "image processing techniques allow extracting".

done

• Line 29: "[11].Note" should be corrected to "[11]. Note". Please check elsewhere in the manuscript.

done

• Line 32: "are" should be corrected to "is".

done

• Lines 43–45: The statement on machine learning is disconnected from the former and subsequent sentences.

The sentence has been removed. Statement on machine learning has been moved after axial transmission signal processing.

• Line 80: "approved 24.01.2017" should be corrected to "approved on the 24.01.2017".

The corrected sentence is:

> no. 16-5714 79 approved **on the** 07.06.**20**16, including an amendment approved **on the** 24.01.2017.

• Line ~~80~~ 100 ?: "is achieved" should be corrected to "are achieved".

The corrected sentence is:

> Finally, four cortical parameters are obtained.

• Line 125: "BMD" should be corrected to "aBMD".

This has been corrected in this paragraph and throughout the text.

• Line 126: "values corresponds" should be corrected to "values correspond".

done

• Line 176: "difference were" should be corrected to "differences were".

done

• Lines 186–188: The statements on the p-value and the VF group should be done after introducing Table 3. Moreover, the latter is introduced before Table 2.

Sentence order has been modified.

• Line 209: "one can observed that the confidence interval are larger" should be corrected to "one can observe that the confidence intervals are larger".

done

• Line 213: "population 1" is misleading as it was never called like this before.

"population 1" has been replaced by "the first population (106 patients)".

• Line ~~213~~ 216 ?: "will discussed" should be corrected to "will be discussed".

done

• Line ~~213~~ 219 ?: "non-fractured and fractures" should be corrected to "non-fractured and fractured".

done

• In Table 3, the statement on the p-values in the legend does not make sense. By the way, this table has room for improvement, as many elements are repeated twice.

The table, now number 4, has been greatly modified.

• Line 228: The abbreviation "aBMD fn" has never been introduced.

The following sentence has been corrected in the paragraph Measurements:

> Three DXA parameters are considered corresponding to measurements performed on the L1–L4 lumbar spine (**a**BMD spine), femoral neck (**a**BMD **fn**) and total femur (**a**BMD **total**).

• Line 258: "lower the DXA" should be corrected to "lower than the DXA".

done

• Line 268: The statement about the multiscale homogenization (Ref. [45]) is disconnected or should be explained better.

The sentence has been removed.

• Line 271: "has" should be corrected to "have".

done

• Line 273: "can be evaluate" should be corrected to "can be evaluated".

done

• Lines 292–293: The statement about cortical bone attenuation should be developed further or removed. Indeed, attenuation seems to be somehow already accounted for in the signal processing to "enhance" the dispersion curves extraction. Incorporating attenuation in the waveguide model would be of course interesting too, but this is not trivial (at least analytically), so please provide some more information thereon and how it may provide an added value towards fracture risk assessment.

The sentence has been modified as

> For example, cortical bone attenuation appears to be a factor of interest [59]. A recent study showed that this parameter could be experimentally estimated using Dictionary Learning and Orthogonal Matching Pursuit points of view [19]. Moreover, attenuation can be taken into the viscoelastic model and therefore into the waveguide model using a simplified homogenization model depending one additional temporal parameter τ^{matrix} [60].

• In Tables 5 and 6, I do think that the term "linear" should be corrected to "logistic".

done

• Please unify the references, as some of them are provided with complete journal names and some others with abbreviated ones, etc.

done

Reviewer 2

The manuscript presents the results of a clinical study on the use of quantitative ultrasound for the detection of osteoporosis. The manuscript is well written and easy to understand. The manuscript focuses on the statistics extracted from the clinical study with limited details on the method the authors used. I understand that the method was already published in other papers but it would be useful to the reader to have a better description of the BDAT method. However, as a scientist with minimal knowledge in statistics, I can hardly judge the quality of the analysis of the results.

There are still a few points that I believe could improve the quality of the manuscript:

1. PLOS Data Policy requires author to make all data available. In this case, the authors say that the data is included in the manuscript. However, I believe that there would be value to the scientific community to be able to access the raw data (time traces) required to extract the bone parameters.

An sentence has been added at the end of the document indicating that raw data are available upon requests to the authors.

2. The current figure resolution is low. The quality should be improved before publication.

Figure quality has been improved.

I finally noted a couple of typos:

line 46 - slower instead of slowest

The sentence has been corrected as

> Authors proposed to measure a second **clinical AT parameter, the** slowest velocity denoted fundamental flexural guided waves (FFGW) or the fundamental anti-symmetric Lamb mode A0.

line 299 - parameters

done

Reviewer 3

The authors evaluated Bi-Directional Axial Transmission (BDAT) device in clinical environment. Four in vivo parameters, including cortical thickness, cortical porosity, velocity of first arriving signal and velocity of A0 mode, are provided by the BDAT device used by the trained operators. The results suggest that the device is affordable and transportable for routine use. Generally, the paper is well organized. The results are solid and convincing. Therefore, the reviewer would recommend acceptance of this manuscript after minor revision. Below are some suggestions for the authors to improve the manuscript.

1. In page 3 line 91, what is the pitch size of the ultrasound probe?

The following sentence has been added

> The transmitter and receiver array pitches are respectfully equal to 1 and 0.8 mm.

2. In page 4 line 139, the author claim that the variable selection was found by subwindow permutation analysis using 1000 Monte Carlo samplings until a stable set was found. Can this part be explained more detailed?

Authors would like to apologize for the confusion. Initially tested, Monte Carlo point of view was not finally used in this study. Only significant parameters were used to build the discrimination model. The corrected sentence is:

> Significant parameters were used to build a first set of discrimination models used to predict vertebral, non-vertebral, and all fragility fractures. A second set of models was also build using parameters associated with p-values lower than 0.01. In addition to DXA and/or BDAT parameters, other parameters were anthropometric data (weight, height, BMI), gender and age.

3. In page 5 line 170, the author claims that the total is 9 hip fractures, but 8 femoral neck, 2 pelvis and 1 pertrochanter are 11 hip fractures. Please check the numbers again.

The corrected sentence is

> Among the 106 patients for whom the measurements were successful, 79 belongs to the 167 control group without fracture (NF), 27 with non-traumatic fractures (F) corresponding 168 to 13 patients with vertebral fractures (VF) and 14 with non vertebral fractures (NVF) associated with the following sites: **10** hip fractures, femoral neck (n = **7**), pelvis (n = 2), pertrochanter (n = 1) as well as 4 others sites, humerus (n = 2), elbow (n = 1) and shoulder (n = 1).

4. In page 2 line 43, some representative approaches in processing axial transmission signals can be considered to be listed before machine learning approaches, such as the Radon transform for multichannel guided waves signal processing and dispersive Radon transform method etc.

The following sentences have been added in the paragraph of the Introduction:

> Several signal processing approaches have been proposed to analyse GW measured with AT, such as the Radon transform for multichannel signals [35], dispersive Radon transform [36], adaptive array signal processing [37] or rotation invariant technique [38]. Recently, Machine and Deep Learning approaches, have been proposed to AT, with or without the need of waveguide modeling [7, 39, 40].

Journal Requirements

When submitting your revision, we need you to address these additional requirements.

1. Please ensure that your manuscript meets PLOS ONE's style requirements, including those for file naming. The PLOS ONE style templates can be found at

$plosone/s/file?id = wjVg/PLOSOne_formatting_sample_main_body.pdf$ and

$plosone/s/file?id = ba62/PLOSOne_formatting_sample_title_authors_affiliations.pdf$.

2. Thank you for stating in your Funding Statement:

[This work was supported by the EFRE.NRW program OsteoSys [EFRE-0800411 and EFRE-0800427, LS-1-1-019c]. Jean-Gabriel Minonzio is supported by Grant ANID / FONDECYT / REGULAR / 1201311 and ECOS 200061. The funders had no role in study design, data collection and analysis, decision to publish, or preparation of the manuscript."

The sentence "*The funders had no role in study design, data collection and analysis, decision to publish, or preparation of the manuscript*" has been added in the Funding Statement.

Please provide an amended statement that declares **all** the funding or sources of support (whether external or internal to your organization) received during this study, as detailed online in our guide for authors at

plosone/s/submit-now.

Please also include the statement "There was no additional external funding received for this study." in your updated Funding Statement.

The sentence "*There was no additional external funding received for this study*" has been added in the Funding Statement.

Please include your amended Funding Statement within your cover letter. We will change the online submission form on your behalf.

3. Thank you for stating the following in the Competing Interests section:

"JGM is a cofounder of Azalée. The remaining authors state that they have no conflicts of interest."

We note that one or more of the authors are employed by a commercial company: Azalée

No authors did receive a salary from Azalée during the study, including preparation, measurement and data analysis.

a. Please provide an amended Funding Statement declaring this commercial affiliation, as well as a statement regarding the Role of Funders in your study. If the funding organization did not play a role in the study design, data collection and analysis, decision to publish, or preparation of the manuscript and only provided financial support in the form of authors' salaries and/or research materials, please review your statements relating to the author contributions, and ensure you have specifically and accurately indicated the role(s) that these authors had in your study. You can update author roles in the Author Contributions section of the online submission form.

Please also include the following statement within your amended Funding Statement.

"The funder provided support in the form of salaries for authors [insert relevant initials], but did not have any additional role in the study design, data collection and analysis, decision to publish, or preparation of the manuscript. The specific roles of these authors are articulated in the 'author contributions' section."

If your commercial affiliation did play a role in your study, please state and explain this role within your updated Funding Statement.

The sentence "*The funders had no role in study design, data collection and analysis, decision to publish, or*

preparation of the manuscript" has been added in the Funding Statement.

b. Please also provide an updated Competing Interests Statement declaring this commercial affiliation along with any other relevant declarations relating to employment, consultancy, patents, products in development, or marketed products, etc.

The sentence "Azalée has currently no commercialisation plan in development."

Within your Competing Interests Statement, please confirm that this commercial affiliation does not alter your adherence to all PLOS ONE policies on sharing data and materials by including the following statement: "This does not alter our adherence to PLOS ONE policies on sharing data and materials." (as detailed online in our guide for authors http://journals.plos.org/plosone/s/competing-interests) . If this adherence statement is not accurate and there are restrictions on sharing of data and/or materials, please state these. Please note that we cannot proceed with consideration of your article until this information has been declared.

Please include both an updated Funding Statement and Competing Interests Statement in your cover letter. We will change the online submission form on your behalf.

4. Thank you for stating the following in the Acknowledgments Section of your manuscript:

" This work was supported by the EFRE.NRW program OsteoSys [EFRE-0800411 and 305 EFRE-0800427, LS-1-1-019c]. Jean-Gabriel Minonzio is supported by Grant ANID / FONDECYT / REGULAR / 1201311 and ECOS 200061. The authors would like to thanks Azalée and BleuSolid for their support"

We note that you have provided additional information within the Acknowledgements Section that is not currently declared in your Funding Statement. Please note that funding information should not appear in the Acknowledgments section or other areas of your manuscript. We will only publish funding information present in the Funding Statement section of the online submission form.

Please remove any funding-related text from the manuscript and let us know how you would like to update your Funding Statement. Currently, your Funding Statement reads as follows:

"This work was supported by the EFRE.NRW program OsteoSys [EFRE-0800411 and EFRE-0800427, LS-1-1-019c]. Jean-Gabriel Minonzio is supported by Grant ANID / FONDECYT / REGULAR / 1201311 and ECOS 200061. The funders had no role in study design, data collection and analysis, decision to publish, or preparation of the manuscript."

Please include your amended statements within your cover letter; we will change the online submission form on your behalf.

The section title "Acknowledgements" has been modified as "Funding Statements".

5. We noticed you have some minor occurrence of overlapping text with the following previous publication(s), which needs to be addressed:

- ieeexplore.ieee.org/document/8579872

D. Ramiandrisoa, S. Fernandez, C. Chappard, M. Cohen-Solal and J. Minonzio, "In Vivo Estimation of Cortical Thickness and Porosity by Axial Transmission: Comparison with High Resolution Computed Tomography," 2018 IEEE International Ultrasonics Symposium (IUS), 2018, pp. 1-4, doi: 10.1109/ULTSYM.2018.8579872.

The text that needs to be addressed involves the Introduction.

In your revision ensure you cite all your sources (including your own works), and quote or rephrase any duplicated text outside the methods section. Further consideration is dependent on these concerns being addressed.

The Introduction have been greatly rewritten.
